# A Network Pharmacology-Based Approach to Investigating the Mechanisms of Fushen Granule Effects on Intestinal Barrier Injury in Chronic Renal Failure

**DOI:** 10.1155/2021/2097569

**Published:** 2021-03-05

**Authors:** Miaoru Han, Hangxing Yu, Kang Yang, Panying Liu, Haifeng Yan, Zhihua Yang, Hongtao Yang

**Affiliations:** Department of Nephrology, First Teaching Hospital of Tianjin University of Traditional Chinese Medicine, Tianjin, China

## Abstract

**Purpose:**

Fushen Granule (FSG) is a Chinese medicine prepared by doctors for treating patients with chronic renal failure, which is usually accompanied by gastrointestinal dysfunction. Here, we explore the protective effect of FSG on intestinal barrier injury in chronic renal failure through bioinformatic analysis and experimental verification.

**Methods:**

In this study, information on the components and targets of FSG related to CRF is collected to construct and visualize protein-protein interaction networks and drug-compound-target networks using network pharmacological methods. DAVID is used to conduct gene ontology (GO) enrichment analysis and Kyoto Encyclopedia of Genes and Genomes (KEGG) pathway enrichment analysis. Then, it is validated by in vitro experiments. In this study, the human intestinal epithelial (T84) cells are used and divided into four groups: control group, model group, FSG low-dose group, and FSG high-dose group. After the experiment, the activity of T84 cells is detected by a MTT assay, and the expressions of tight junction protein ZO-1, claudin-1, nuclear factor erythroid 2-related factor (Nrf2), heme oxygenase*-*1 (HO-1), malondialdehyde (MDA), and cyclooxygenase*-*2 (COX-2) are examined by immunofluorescence and/or western blotting.

**Results:**

Eighty-six potential chronic renal failure-related targets are identified by FSG; among them, nine core genes are screened. Furthermore, GO enrichment analysis shows that the cancer-related signaling pathway, the PI3K-Akt signaling pathway, the HIF1 signaling pathway, and the TNF signaling pathway may play key roles in the treatment of CRF by FSG. The MTT method showed that FSG is not cytotoxic to uremic toxin-induced injured T84 cells. The results of immunofluorescence and WB indicate that compared with the control group, protein expressions level of ZO-1, claudin-1, and Nrf2 in T84 cells is decreased and protein expressions level of HO-1, MDA, and COX-2 is increased after urinary toxin treatment. Instead, compared with the model group, protein expressions level of ZO-1, claudin-1, and Nrf2 in T84 cells is increased and protein expressions level of HO-1, MDA, and COX-2 is decreased after FSG treatment.

**Conclusion:**

FSG had a protective effect on urinary toxin-induced intestinal epithelial barrier injury in chronic renal failure, and its mechanism may be related to the upregulation of Nrf2/HO-1 signal transduction and the inhibition of tissue oxidative stress and inflammatory responses. Screening CRF targets and identifying the corresponding FSG components by network pharmacological methods is a practical strategy to explain the mechanism of FSG in improving gastrointestinal dysfunction in CRF.

## 1. Introduction

Chronic kidney disease (CKD) is a kind of renal disease featuring chronic renal dysfunction and structural damage. The glomerular filtration rate (GFR) is the best index reflecting overall kidney function [1]. CKD has become a serious global public health problem. There were 1.2 million deaths worldwide in 2017 due to CKD. The mortality of CKD has been rising, and it is predicted that, by 2040, the number will rise worldwide to 2.2 million conservatively, even up to 4 million [2, 3]. The renal function of CKD patients gradually decreases due to nitrogen waste accumulation, which is discharged by normal persons over time and eventually progresses to chronic renal failure (CRF), which is always accompanied by a progressive decline in glomerular filtration rate and the continuous accumulation of metabolic waste. In recent years, a large number of studies have reported that the accumulation of urinary toxins and the influx of other residual toxins destroy the intestinal epithelial barrier, allowing the entry of intestinal bacteria into the blood, which further aggravates the progression of CRF [4, 5], and finally, end-stage renal disease (ESRD) develops. The gut barrier is composed of occludin, ZO-1, and E-cadherin. The expression of claudin-1, occludin, and ZO-1 in the intestinal tissue of five out of six nephrectomized rats was significantly reduced, and there was significant systemic oxidative stress in the rats [6]. In addition, the content of enterotoxins such as para-cresol sulfate (PCS) and indole sulfate (IS) was significantly increased in the serum of CKD patients and long-term dialysis patients, indicating increased intestinal permeability [7, 8]. In CKD and long-term dialysis patients with damaged intestinal epithelial barriers, intestinal bacterial DNA and uremia-related toxins infiltrate the blood from the intestinal tract, and white blood cells in the blood are activated. This results in the generation of local intestinal cytokines, and further contractions or tight junction protein reactions between endocytic cells occur [9]. This process is a vicious circle, which eventually leads to chronic systemic inflammation. Studies have shown that bacterial DNA fragments from the gut can be detected in the serum of CKD patients whether they are nondialysis patients or dialysis patients [8, 10]. Therefore, the level of endotoxin from the gut flora in patients with CFR is significantly related to intestinal dysfunction. Lin [11] found that the total serum Is and PC levels in patients with advanced CKD were related to aortic calcification, increased vascular stiffness, and the risk of cardiovascular death. Kim [12] found that the elimination of uremic toxins was an effective treatment for low-turnover osteopathy in CKD patients. Therefore, an important way to improve the nutritional status and quality of life of uremic patients is to regulate intestinal dysfunction. The main symptoms of CRF intestinal injury are abdominal distention, abdominal pain, diarrhea, and constipation. In clinical treatment, antibiotics and gastrointestinal motility drugs are often used. However, the improvement of intestinal mucosal injury by these drugs has not been confirmed, and there is no specific drug to prevent or inhibit intestinal barrier injury.

In China, traditional Chinese medicine is often used to regulate gastrointestinal function and has shown significant effects [13]. Compared to single structure drugs, traditional Chinese medicine has the advantages of multidirectional and multitarget regulation in disease treatment [14]. Through long-term clinical practice, our team found that the traditional Chinese medicine Fushen Granule (FSG) significantly improved the condition of CRF patients complicated with intestinal symptoms [15]. The components of FSG include Salviae miltiorrhizae Radix et Rhizoma (Danshen in Chinese, 30 g), Citri Reticulatae Pericarpium (Chenpi in Chinese, 10 g), Astragali Radix (Huangqi in Chinese, 15 g), Pinelliae Rhizoma (Banxia in Chinese, 15 g), root of Chinese clematis (Weilingxian in Chinese, 15 g), Radix et Rhizoma Rhei (Shoujun in Chinese, 10 g), Euonymus alatus (Guijianyu in Chinese, 30 g), and *Angelica sinensis* (Danggui in Chinese, 10 g). Previous studies have shown that FSG could significantly improve the permeability of the intestinal barrier in peritoneal dialysis rats with CRF. In addition, it also delayed the removal of urinary toxins and water from peritoneal dialysis patients, protected the residual renal function, and improved the nutritional status and clinical syndrome characteristics of peritoneal dialysis patients [16]. Since FSG contains hundreds of compounds and acts on many cell targets, it is difficult to systematically study the mechanism of FSG using conventional methods. Therefore, this study used a network pharmacology strategy to analyze the main active components of FSG from the perspective of a traditional Chinese medicine-compound gene system and explored the mechanism of FSG with multiple components and multiple targets in treating CRF. Then, we subjected the analysis results to experimental verification. In this study, human colon epithelial cells (T84) were cultured *in vitro* to establish an intestinal barrier injury model induced by urotoxin in CRF and further observe the mechanism of FSG intervention on gut dysfunction induced by urotoxin in CRF.

## 2. Materials and Methods

### 2.1. Prediction of Traditional Chinese Medicine Targets and Corresponding Active Ingredients

The chemical constituents of Huangqi, Danggui, Xianlingpi, Chenpi, Banxia, Shujun, Danshen, and Guijianyu were identified using the Chinese Medicine System Pharmacology Database and Analysis Platform (TCMSP) and related literature (http://tcmspw.com/tcmsp.php) [17]. On this basis, this study combined the oral bioavailability (OB ≥30%) of chemical ingredients and drug-likeness (DL ≥0.18) as the criteria for screening the active compounds in FSG and identifying the target protein of the corresponding compound. The obtained target protein name was input into the Uniprot (https://www.uniprot.org/) database [18], the species was limited to humans, and all the input target names were corrected to the gene name of the target for the subsequent related analysis.

### 2.2. Prediction of Chronic Renal Failure-Related Targets

The National Center for Biotechnology Information (NCBI) (https://www.ncbi.nlm.nih.gov/) [19] database is used to provide information on drugs, targets, and drug-targeted diseases and pathways. The Diskenet (https://www.diskenet.org/) [20] database is a comprehensive gene disease association (GDA) relational database. It provides the latest information on human diseases. The Drugbank (https://www.drugbank.ca/) [21] database provides comprehensive information about western medicine molecules and their mechanisms, including their chemistry, pharmacology, interaction information, and targets. The Therapeutic Target Database (TTD) (http://db.idrblab.net/ttd/) [22] provides information on drugs, targets, diseases, and pathways targeted by drugs. In these four databases, “chronic renal failure” was entered to obtain CRF-related target genes.

### 2.3. Intersection of Drug Targets and Disease Targets

A Venn diagram (http://bioinformatics.psb.ugent.be/webtools/Venn/) was drawn, and Wayne diagram software was used to analyze the two datasets online to find the intersecting genes in the two datasets.

### 2.4. Construction of Drug-Target-Disease Interaction Network

The Protein-Protein Interaction Networks Functional Enrichment Analysis (STRING) database provides information on predicted and experimental protein-protein interactions [23]. STRING online (https://string-db.org/cgi/input.pl) was used to construct a protein-protein interaction (PPI) network from the intersection of drug targets and disease targets. The database uses the confidence range of the data scores to define PPI (low confidence is defined by scores of >0.15; medium confidence by scores of >0.4; and high confidence by scores of >0.7). Then, the Merge function of Cytoscape software (Cytoscape_v3.2.1) [24] was used to construct a traditional Chinese medicine-compound-target network: (1) the intersecting gene and the corresponding Chinese medicine compound were used to establish a PPI network of the Chinese medicine compound; (2) a traditional Chinese medicine-target PPI network was constructed by connecting the known intersecting genes with their corresponding traditional Chinese medicine; (3) the two networks (1) and (2) were intersected to construct a drug-compound-target pathway network.

### 2.5. Gene Ontology Analysis

Gene ontology (GO) analysis was carried out for the intersecting genes of the drug targets and disease targets using the Database for Annotation, Visualization, and Integrated Discovery (DAVID) database (https://david.ncifcrf.gov/). The DAVID database integrates various types of database resources. The improved Fisher accurate test algorithm was used to analyze the function and path enrichment of differential genes in the gene set, and the *P* value and false discovery rate (FDR) were provided for the rich set analysis results [25]. GO enrichment analysis clarifies the role of traditional Chinese medicine compound-target proteins in gene function, including the function of three modules: biological processes (BP), molecular function (MF), and cell composition (CC) [26].

### 2.6. Kyoto Encyclopedia of Genes and Genomes Pathway Enrichment Analysis

Similarly, through the DAVID database (https://david.ncifcrf.gov/) for the enrichment analysis of drug-disease intersection protein targets, KEGG pathway enrichment analysis not only provides an annotation of pathway functions for a given gene set but also provides pathway enrichment analysis [27]. For the multiple test correction of the significant *P* value in enrichment analysis, the enrichment analysis adopts the algorithm designed by the hypergeometric algorithm and uses the Benjamini–Hochberg correction method. Finally, through the role of the target in the pathway connections, the relevant pathway is obtained [28].

## 3. Experimental Verification

### 3.1. Experimental Materials and Methods

#### 3.1.1. Experimental Cell Lines

The human colon epithelial T84 cell line was purchased from the American Standard Biological Collection Center ATCC (LOT: 62783415).

#### 3.1.2. Experimental Animals

Specific pathogen-free (SPF) male Sprague-Dawley (SD rats), weighing 180–220 g, were purchased from Beijing Viton Lihua Experimental Animal Technology Co., Ltd.

#### 3.1.3. Drug Preparation

FSG was composed of Huangqi 15 g, Danggui 10 g, Xianlingpi 15 g, Chenpi 10 g, Banxia 15 g, Danshen 30 g, Shuju 10 g, and Guijianyu 30 g. The abovementioned traditional Chinese medicine decoction-free granules were provided by the First Affiliated Hospital of Tianjin University of Traditional Chinese Medicine.

#### 3.1.4. Preparation of FSG Drug-Containing Serum

Eighty SD rats were adaptively fed for one week and randomly divided into three groups: the blank serum group, the low-dose FSG intragastric group, and the high-dose FSG intragastric group. According to the clinical equivalent dose between humans and rats, the rat dose was calculated as 9 mL kg^−1^ d^−1^. The low dose was given as 4.5 mL kg^−1^d^−1^, and the high dose was 18 mLkg^−1^d^−1^. The blank serum group was gavaged with normal saline once a day for seven consecutive days with free access to diet. Anesthesia was performed 2 hours after the last gavage. Blood, collected from abdominal aorta of anesthetized rats after 2 hours, was centrifuged to get supernatant. The complement was inactivated by water bath at 56°C for 30 min, followed by removal of bacteria through microporous filtration membrane, and then the serum was packed and frozen at −80°C.

#### 3.1.5. Cell Culture Treatments

Human colon epithelial T84 cells were cultured in 50 U L^−1^ penicillin streptomycin^−1^ Dulbecco's modified Eagles Medium (DMEM)/F12 medium containing 10% fetal bovine serum (FBS; Hyclone, USA) and 50 U L^−1^ penicillin and streptomycin in 5% CO_2_ and 37°C constant temperature conditions. The cell media were changed every one to two days. Some of the cells grown to the logarithmic phase were subcultured, and the other cells were frozen for future use.

#### 3.1.6. Experimental Model

The human intestinal epithelial T84 cells were cultured *in vitro* to a confluent state, and different concentrations of urea (0 mg dL^−1^, 72 mg dL^−1^, and 144 mg dL^−1^) and different durations (24 h and 48 h) were used to treat the T84 cells to simulate intestinal epithelial barrier injury. The effect of different concentrations of FSG on the viability of T84 cells was detected by the methyl thiazolyl tetrazolium (MTT) method, and the concentration of urea used for modeling was finally determined.

#### 3.1.7. Experimental Grouping

The cell treatment groups were as follows:

  ① Control group (group C): DMEM/F12 supplemented with 10% serum
  ② Model group (group M): DMEM/F12 supplemented with 10% serum and 144 mg dL^−1^ urea
  ③ Low-dose FSG-containing serum group (FSG low-dose group): DMEM/F12 supplemented with 144 mg dL^−1^ urea and 10% serum containing low-dose FSG
  ④ High-dose FSG-containing serum group (FSG high-dose group): DMEM/F12 supplemented with 144 mg dL^−1^ urea and 10% serum containing high-dose FSG

### 3.2. Detection

#### 3.2.1. MTT Assay to Detect the Effect of Different Concentrations of FSG on the Viability of T84 Cells

T84 cells were cultured and collected, followed by 0.25% trypsin digestion and Pekin dilution. The cell concentration was adjusted to 50,000 cells mL^−1^ and plated in 96-well plates. Every well contained 100 *μ*L, with six double wells per group and incubated at 5% CO_2_ at 37°C. Until the cells grew to confluence, different concentrations of urea (42 mg dL^−1^, 72 mg dL^−1^, and 144 mg dL^−1^) and 10% serum containing low-dose FSG or high-dose FSG were added and further incubated in a 5% CO_2_ environment at 37°C for 24 h. MTT at 5 mg mL^−1^ or 0.5% was added to each well, followed by incubation at 37°C for another 4 h in a ventilated environment, and then, the supernatant was replaced with 150 *μ*L of dimethyl sulfoxide (DMSO) to fully dissolve the crystals with low-speed shaking for 10 min. The absorbance was determined at 490 nm with an enzyme-linked immunoassay detector. Cell viability was calculated by the following formula: cell viability of control = (medicine group A value − zero hole A value)/(control well A value − zero hole A value) × 100%.

#### 3.2.2. Cell Immunofluorescence Staining to Detect the Expression and Distribution of ZO-1 and Claudin-1

The logarithmic-phase cells were collected, digested with 0.25% pancreatin, diluted with culture medium, adjusted to a concentration of 1 × 10^6^ cells on cell-climbing tablets in 24-well plates. Those cells, which were divided into the blank group, the model group (144 mg dL^−1^ urea), the low-dose group of traditional Chinese medicine (FSG low-group), and the high-dose group of traditional Chinese medicine (FSG high-group), were treated with medicine and incubated at 37°C in 5% CO2 until the cells grew to fusion. After incubation at 37°C for 24 h, the cells were fixed with 4% paraformaldehyde (PA) for 20–30 minutes at room temperature. Then, the cells were washed with phosphate-buffered saline (PBS) twice for 5 minutes and treated with 0.5% Triton 100 for 10 minutes. After washing with PBS three times for 5 min at room temperature, the cells were incubated in 5% goat serum blocking solution at room temperature for 1 h and the cells were washed with PBS again. Rabbit anti-ZO-1 and claudin-1 (1 : 100 dilution) were added to the cells and incubated overnight at 4°C.

The next day goat anti-rabbit IgG (1 : 200) was added and incubated in the dark at 37°C for 2 h, after washing by PBS for 5 min three times. Then, the samples were washed again. 4′,6-Diamidino-2-phenylindole (DAPI) antifluorescence quenching agent was added and incubated for 1 min, and the slides were sealed and imaged under a laser confocal microscope.

#### 3.2.3. Western Blots Were Used to Detect the Expression of ZO-1, Claudin-1, Nrf2, HO-1, MDA, and COX-2

T84 cells in the logarithmic growth stage were cultured synchronously and collected 48 h after treatment with 144 mg dL^−1^ urea and serum containing low and high dosages of FSG. The total protein was extracted and by lysing in RIPA buffer (containing 1% proteinase inhibitor and phosphatase inhibitor). The protein concentration was determined by the BCA Protein Assay Kit (Boster, China). Protein (10 *μ*gmL^−1^) was separated on 10% sodium dodecyl sulfate (SDS)-polyacrylamide gel electrophoresis (PAGE), at 80 V constant voltage for 30 min, 140 V constant voltage for 40 min, and a constant current of 200 mA for wet conversion for 2 h; the proteins were transferred to a polyvinylidene fluoride (PVDF) membrane. After being transferred, the internal reference and target bands were cut according to the molecular weight of 200 mA. After incubation, the corresponding antibody was incubated at 4°C overnight. After washing once with Tris-buffered saline and Tween (TBS-T) for 10 min three times, horseradish peroxidase (HRP)-labeled goat anti-rabbit or goat anti-mouse secondary antibody was added and incubated for 1 h at room temperature. After washing with TBST three times for 10 min, enhanced chemiluminescence developer was added at a ratio of 1 : 1 to react for 1 min, and the membrane was put it into a gel imaging system for development.

#### 3.2.4. Statistical Analysis

Statistical analysis was performed by SPSS software v25.0. Data were presented as mean ± standard error (SE). Multiple comparisons were done by one-way analysis of variance (ANOVA) followed by the Turkey multiple comparisons test. In the case of nonnormally distributed data, the Kruskal–Wallis test was used. A *P* value <0.05 was considered statistically significant.

## 4. Results

### 4.1. Prediction of Traditional Chinese Medicine Targets and the Corresponding Active Ingredients

Each FSG component was input into the TCMSP for screening. Oral bioavailability (OB ≥30%) and drug similarity (DL ≥0.18) were used as screening criteria. Finally, the screened target proteins were imported into UniProt and converted into gene names. A total of 2532 target genes were retrieved. Among them, 866 were associated with Danshen, 69 with Chenpi, 447 with Huangqi, 68 with Danggui, 511 with Yinyanghuo, 284 with Guijianyu, 173 with Banxia, and 114 with Dahuang. After removing the duplicates, a total of 378 genes were collected.

### 4.2. Prediction of Targets Related to Chronic Renal Failure

The disease genes were searched in four databases: the NCBI (https://www.ncbi.nlm.nih.gov/), DisGeNET (https://www.disgenet.org/), Drugbank (https://www.drugbank.ca/), and the TTD (http://db.idrblab.net/ttd/) by entering “chronic renal failure” and limiting the species to humans. Finally, a total of 1327 genes were retrieved. Among them, NCBI retrieved a total of 63 genes, DisGeNET retrieved a total of 1251 genes, Drugbank retrieved a total of 16 genes, and TTD retrieved a total of one gene. After removing the duplicates, a total of 666 genes were collected.

### 4.3. Acquisition of Intersecting Genes

**Figure 1:**
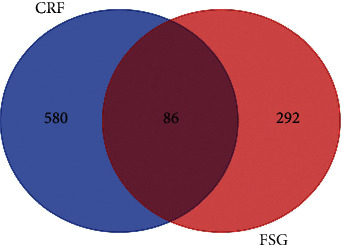
Eighty-six targets are common to FSG and CRF.

The potential gene targets of CRF-related genes and FSG active ingredients were used to draw a Venn diagram (http://bioinformatics.psb.ugent.be/webtools/Venn/) and the potential target genes in which FSG played an important role in CRF were obtained. The Venn diagram showed a total of 86 overlapping genes (Figure 1).

### 4.4. Construction and Analysis of PPI Network

**Figure 2:**
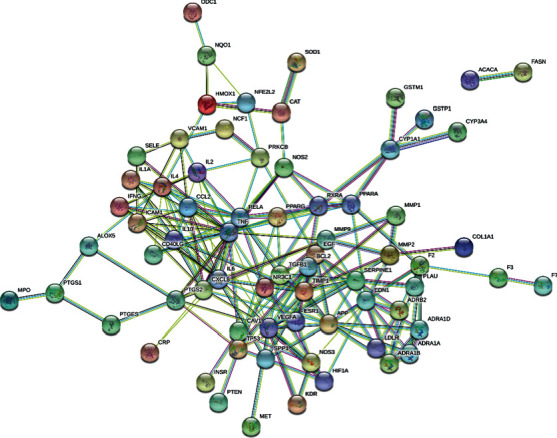
PPI network built by the STRING online database and module analysis.

**Figure 3:**
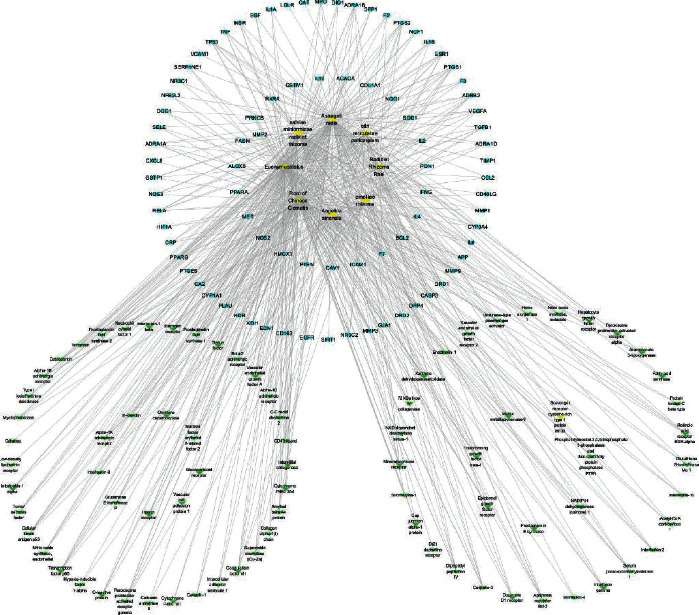
Drug-compound-target network. Yellow represents the drugs, blue represents the target genes, green represents the compounds, and the edge line represents the relationship between them.

Since proteins often form macromolecular complexes through interactions, they can complete biological functions in cells [26]. To further explore the mechanism of FSG in treating CRF, we submitted the intersecting genes to STRING software to construct a PPI network and screened out target protein interaction data with confidence scores of >0.9. A total of 70 core target proteins were screened (Figure 2). Then, we applied Cytotype MCODE v 1.4.1 for further analysis, and the results showed that 17 central nodes were identified among the 70 nodes (Figure 3). Then, the intersecting genes were imported into Cytoscape software, the parameters were adjusted, and the results are shown in Figure 3. The network consisted of 174 nodes and 600 edges. In addition, we screened nine core genes based on the degree (Table 1). These genes are involved in various pathogenic CRF processes, including inflammation and fibrosis.

### 4.5. GO and KEGG Pathway Analysis of Target Proteins

**Figure 4:**
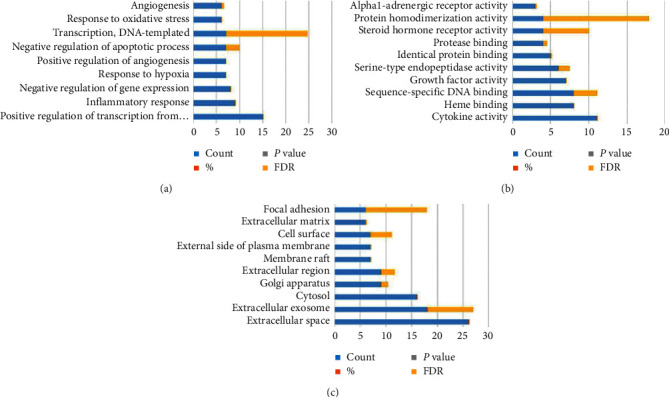
(a). Analysis of 10 biological processes of the core targets of the effect of Fushen Granule (FSG) on chronic renal failure (CRF). (b) Analysis of 10 molecular functions of the core targets of the effect of FSG on CRF. (c) Analysis of 10 cell components of the core target of the effect of FSG on CRF.

To further clarify the biological role of these genes, GO function analysis of the intersecting genes was conducted in the DAVID database (https://david.ncifcrf.gov/) with significance indicated by a *P* value of <0.01. The results showed the following: (1) for BP, position regulation of transcription from the RNA polymer II promoter, informational response, and negative regulation of gene expression were identified; (2) for MF, the intersecting genes were mainly enriched in the negative regulation of gene expression, heme binding, and sequence-specific DNA binding; (3) for the CCs, the 5 intersecting genes were mainly enriched in extractor exosomes, cytosol, and Golgi apparatuses (Table 2 and Figure 4).

**Figure 5:**
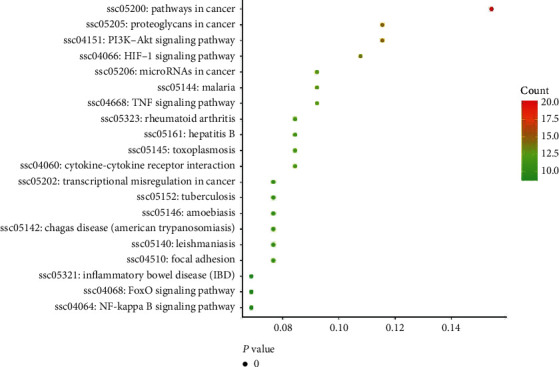
Enrichment analysis of candidate targets for FSG against CRF.

To understand the action pathway of the 86 selected intersecting genes, KEGG pathway enrichment was analyzed in the DAVID database. KEGG enrichment was measured by gene number, *P* value (*P* < 0.01), and enrichment factor. The results showed that these genes were significantly enriched in 61 pathways related to cancer, proteoglycans in cancer, the PI3K-Akt signaling pathway, and others. Here, we list only the top 20 pathways (Table 3 and Figure 5).

### 4.6. Experimental Verification

#### 4.6.1. FSG Has a Nontoxic Effect on Uremic Toxin-Induced T84 Cells

**Figure 6:**
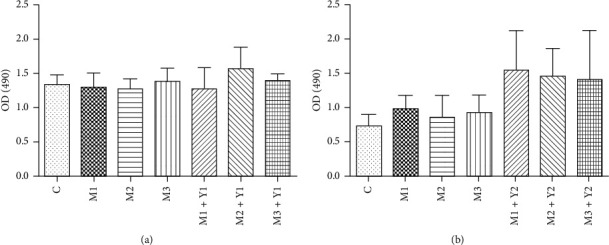
Effect of various concentrations of uremic toxin and low (a) and high-dose (b) FSG-containing serum on T84 viability.

The MTT experiment showed that treatment with 42 mg dL^−1^, 72 mg L^−1^, and 144 mg L^−1^ urotoxin did not affect the viability of T84 cells (*P* > 0.05). Therefore, we selected 144 mg L^−1^ urotoxin for modeling. The cells were treated with low-dose FSG-containing serum and high-dose FSG-containing serum along with various concentrations of uremic toxin. High-dose FSG had a slight proliferative effect on cells in each urea treatment group, but with no statistical significance (*P* > 0.05; Tables 4 and 5 and Figure 6).

#### 4.6.2. Effect of FSG on the Expression of ZO-1 and Claudin-1 in Urotoxin-Treated T84 Cells

**Figure 7:**
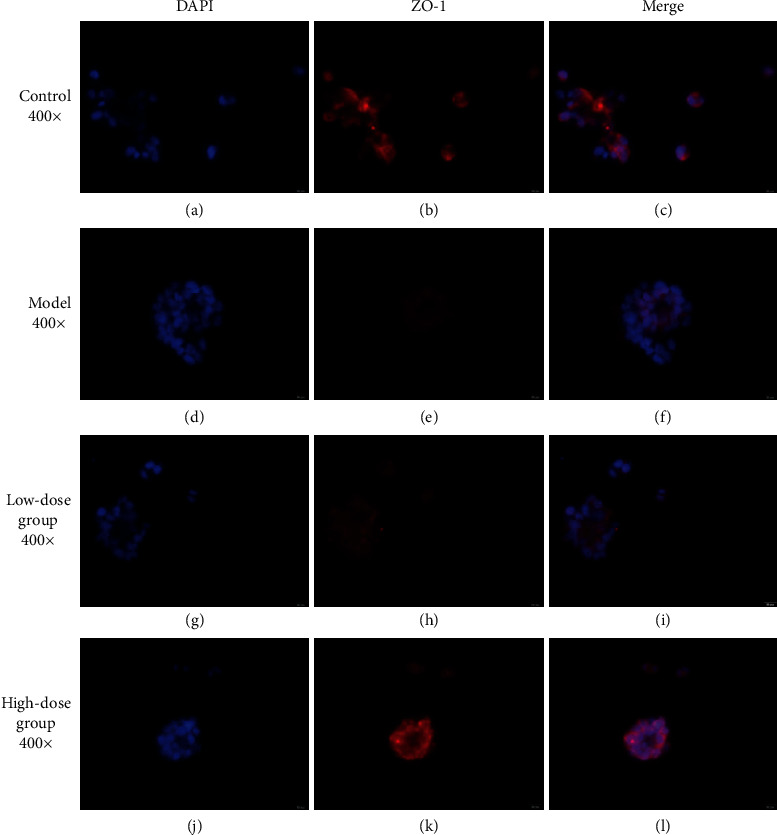
The protein levels of ZO-1 in T84 cells based on immunofluorescence staining.

**Figure 8:**
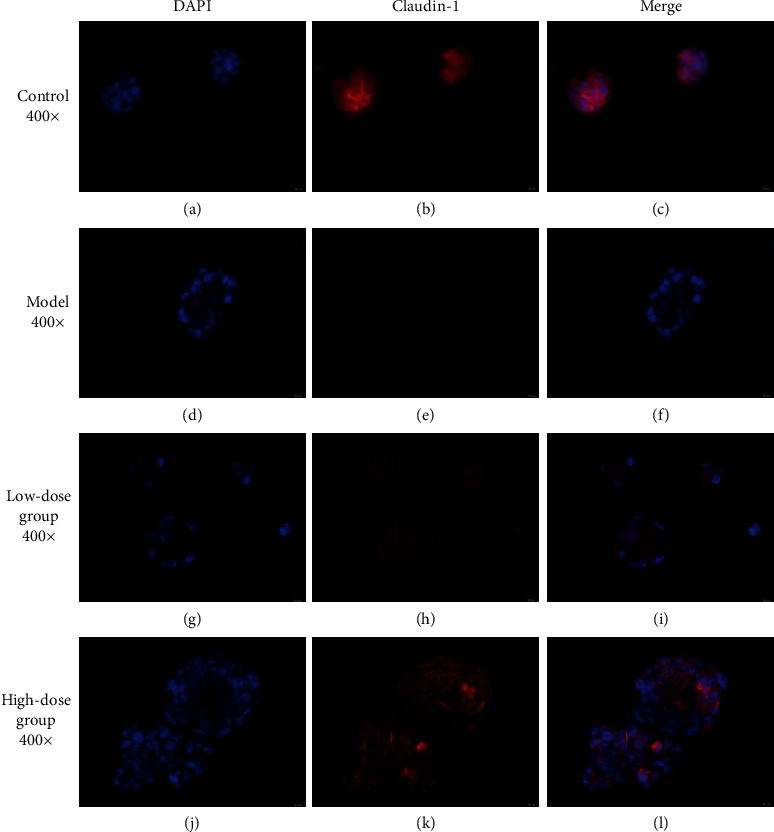
The protein levels of claudin-1 in T84 cells based on immunofluorescence staining.

**Figure 9:**
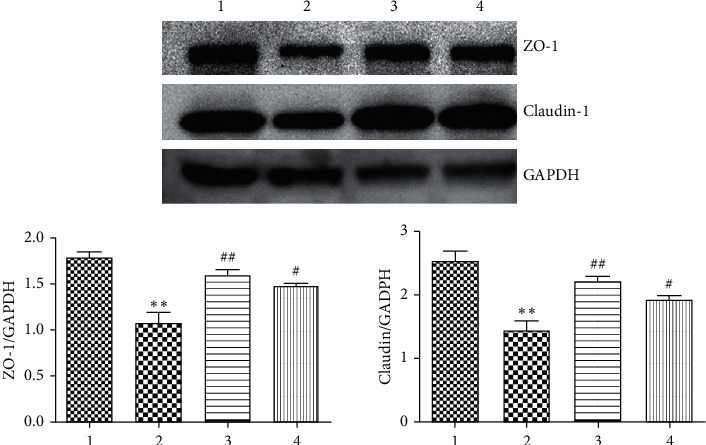
Protein expression of ZO-1 and claudin-1. The data are expressed as the mean ± SE, *n* = 4. 1: group C; 2: group M; 3: FSG low-dose group; 4: FSG high-dose group. Compared to the control group, ^*∗*^*P* < 0.05 and ^*∗∗*^*P* < 0.01; compared to the model group, ^#^*P* < 0.05 and ^##^*P* < 0.01.

Claudin plays an important role in maintaining normal intestinal barrier function. ZO-1 and claudin-1 are two important tight junction proteins [29]. Previous studies have shown that, in animal models and human colon cells cultured *in vitro*, CKD induced a loss of intestinal epithelial tight junction proteins ZO-1 claudin-1 and occludin [6]. To observe the changes in ZO-1 and claudin-1 better, we performed using immunofluorescence staining assays to detect ZO-1 and claudin-1 in the T84 cells of each group. Compared to the blank group, the expression of ZO-1 (red) (Figure 7) and claudin-1 (red) (Figure 8) in the model group decreased, and the expression of ZO-1 and claudin-1 after treatment with low- and high-dose FSG-containing serum increased. According to the results, the model was established successfully. The western blotting results showed compared to the control group, the expression levels of ZO-1 and claudin-1 in the model group decreased (*P* < 0.01). Compared to the model group, the expression levels of ZO-1 and claudin-1 in the low-dose group increased significantly (*P* < 0.01), and the expression levels of ZO-1 and claudin-1 in the high-dose group increased (*P* < 0.05) (Figure 9).

#### 4.6.3. Effect of FSG on Nrf2, HO-1, MDA, and COX-2 of T84 Cells

**Figure 10:**
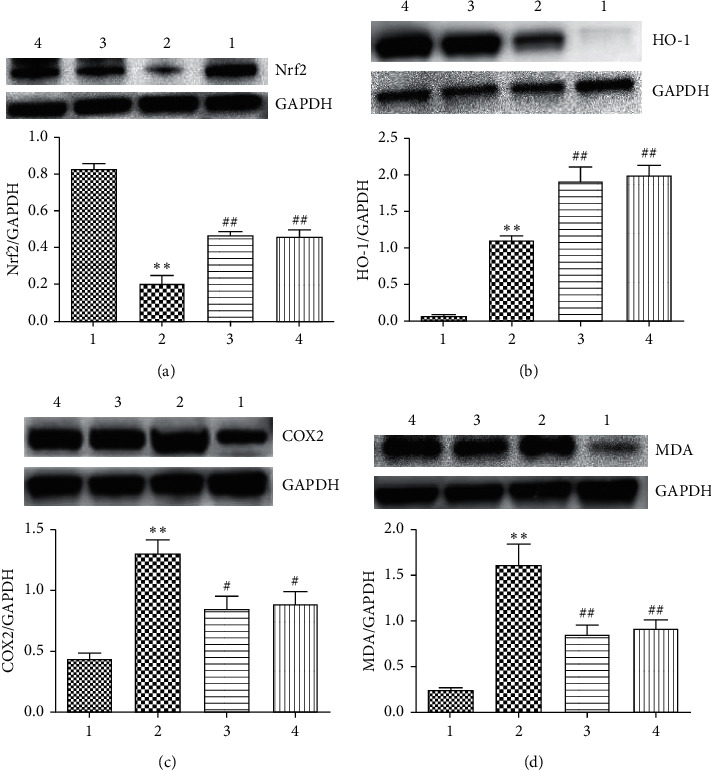
The effect of FSG on T84 intestinal epithelial cells: (a) protein expression of Nrf2; (b) protein expression of HO-1; (c) protein expression of COX-2; (d) protein expression of MDA. The data are expressed as the mean ± SE, *n* = 4; 1, group C; 2, group M; 3, FSG low-dose group; 4, FSG high-dose group. Compared to the control group, ^*∗*^*P* < 0.05 and ^*∗∗*^*P* < 0.01; compared to the model group, ^#^*P* < 0.05 and ^##^*P* < 0.01.

As shown in Figure 10, compared to the control group, the heme oxygenase-1 (HO-1) protein content increased (*P* < 0.01), the Nrf2 total protein content decreased (*P* < 0.01), and Nrf2 and HO-1 protein expression in the group treated with high- and low-dose serum containing FSG increased (*P* < 0.01). Compared to the blank group, the expression level of cyclooxygenase-2 (COX-2) protein and MDA protein in the model group was significantly higher (*P* < 0.01). The expression level of MDA in the groups treated with serum containing FSG at each concentration was increased significantly (*P* < 0.01), and the differences were statistically significant. The expression level of COX-2 in the groups treated with serum containing FSG at each concentration was lower (*P* < 0.05).

## 5. Discussion

### 5.1. Network Pharmacology and FSG

This study systematically explored the pharmacological mechanism of FSG in the treatment of gastrointestinal dysfunction in chronic renal failure through network pharmacological analysis and experimental verification. Because traditional Chinese medicine has a lot of medicinal exerts and complex ingredients, the effective ingredients of many drugs are not clear at present. Thus, it is difficult for traditional research methods to clarify their mechanisms. We analyzed the compound composition of each drug in FSG through network pharmacology and then used these compounds to identify the corresponding target proteins and gene names. The same method was used to find the intersection of target proteins and genes corresponding to CRF, and 86 potential protein targets for treating CRF with FSG were analyzed. Then through the STRING network, we obtained the protein interaction network map.

Cytoscape was used to construct the FSG Chinese medicine-compound-target network diagram, which had 174 nodes and 600 edges. Among the 174 nodes, there were 81 compound nodes, 85 target nodes, and 8 drug nodes. The results showed that FSG had multicomponent, multitarget, and synergistic treatment characteristics. Then, we applied network analysis to screen out 9 core candidates. We determined the 9 core candidate components of FSG in treating CRF according to the degree. PTGS2 (prostaglandin G/H synthase 2), PTGS1 (prostaglandin G/H synthase 1), ADRB2 (beta-2 adrenergic receptor), DPP4 (dipeptidyl peptidase IV), RXRA (retinoic acid receptor RXR-alpha), ADRA1B (alpha-1B adrenergic receptor), PPARG (peroxisome proliferator-activated receptor-gamma), ESR1 (estrogen receptor), and F2 (thrombin) were closely related to the active components of FSG in treating CRF. Among them, PTGS2, also known as COX-2, had the highest intermediate level. COX-2 has very low activity in normal tissue cells, while in inflammatory cells, cytokines and mitogenic factors can significantly induce COX-2 expression [30]. Studies have shown that COX-2 plays an important role in the regulation of various chronic kidney diseases [31]. At the same time, COX-2 plays a key role in regulating renin release and water and salt metabolism and maintaining blood pressure [32, 33]. They interact with each other to delay the progression of CRF through different signaling pathways.

To clarify how FSG worked through these targets, this study further analyzed GO and KEGG pathway enrichment. FSG was found to play anti-inflammatory and antioxidation roles by regulating the activity of some cytokines and growth factors, and FSG also plays an important part in regulating the negative regulation of apoptosis, angiogenesis, RNA regulation, and coping with hypoxia. Signal transmission is mainly on the surface of the cell membrane, Golgi, extracellular matrix, and the outer side of the plasma membrane. The KEGG results showed that FSG may be involved in the cancer signaling pathway, the PI3K-Akt signaling pathway, the HIF1 signaling pathway, and the TNF signaling pathway. The PI3K/Akt signaling pathway is involved in biological activities including apoptosis, metabolism, proliferation, and differentiation. Previous studies have proved that the PI3K/Akt signaling pathway plays an important role in the treatment of renal failure and hypertension [34]. However, the exact mechanism needs further study. Hypoxia-inducible factor 1 (HIF1) is an important factor for maintaining oxygen balance in the body. Under hypoxic conditions, the glomeruli, the capillary network around the renal tubules, and the blood vessels entering and exiting the small arteries contract, resulting in decreased blood flow. This decreases the glomerular filtration rate, and the tubule interstitial becomes hypoxic, which leads to renal fibrosis [30]. Therefore, chronic hypoxia and CRF are linked, and the role of FSG in these processes needs further study. Cancer signaling pathways such as the MAPK signaling pathway [35], NF-*κ*B signaling pathway [36], and Wnt signaling pathway [37] are all linked to chronic renal failure. Mitogen-activated protein kinase (MAPK) is a functionally related kinase that regulates key cellular processes (e.g., survival, death, differentiation, and proliferation) involved in kidney disease [38]. Nuclear factor-kB (NF-KB) is an important nuclear transcription factor in cells. It plays a key role in the regulation of genes such as fibronectin (FN), tumor necrosis factor-*α* (TNF-*α*), interleukin-8 (IL-8), and monocyte chemoattractant protein-1 (MCP-1) related to inflammatory responses, thus aggravating renal damage [39]. The Wnt/*β* catenin signaling pathway is a classical Wnt pathway. The Wnt/*β* catenin signaling pathway was reported to have little activity in normal adult kidneys but was activated after various kidney injuries [40, 41]. Oxidative stress and inflammation also play important roles in cancer signaling pathways [42, 43]. A previous GO enrichment analysis found that FSG had obvious anti-inflammatory and antioxidant effects in the treatment of CRF. Previous studies have shown that FSG could correct intestinal dysfunction in patients with peritoneal dialysis and protect residual renal function [44]. Therefore, through the network pharmacological analysis, experiments on oxidative stress and anti-inflammation were conducted.

### 5.2. Experimental Study of FSG on Chronic Renal Failure Gastrointestinal Dysfunction

Gastrointestinal dysfunction in CRF is very common in patients with CRF and ESRD. The stimulation of the gastrointestinal tract by uremia toxins and some metabolites has long been considered to be an important cause of gastrointestinal dysfunction in CRF [45]. In this study, human colon epithelial cells (T84) were cultured *in vitro* to establish an intestinal barrier injury model of CRF induced by urotoxin. Changes in tight junction proteins ZO-1 and claudin-1, inflammatory factor COX-2, and Nrf2/HO-1 signal transduction in intestinal epithelial cells were observed. The study found that serum containing different concentrations of FSG could upregulate the expression of intestinal epithelial tight junction proteins claudin-1 and ZO-1 to different degrees and played a protective role in the intestinal epithelial barrier of T84 cells. As an inducible enzyme, COX-2 can initiate and regulate the inflammatory response of tissues [46]. This study found that the COX-2 protein in the model group was significantly increased, whereas the COX-2 protein in each FSG treatment group was decreased (*P* < 0.05). FSG has been suggested to improve intestinal epithelial barrier cells injured by urotoxin by inhibiting inflammatory reactions. Nrf2 is the most important transcriptional regulator of oxidative stress and inflammation. A large amount of evidence indicates that Nrf2 protects cells from oxidative stress by activating the expression of genes encoding detoxification, antioxidant, and anti-inflammatory proteins [47]. Under physiological conditions, Nrf2 is isolated in the cytoplasm by Kelch-like ECH-related protein 1 (Keap1). However, when organisms are exposed to oxidative stress or some toxic effects (such as ultraviolet radiation, radiation, and air pollution), Nrf2 is dissociated from Keap1, rapidly translocates into the nucleus, combines with MAF protein to form a dimer, and then combines with the antioxidant response element (ARE), regulating the expression of 65 antioxidant genes, including NADPH quinone oxidoreductase 1 (NQO1) and HO-1 [48–50]. This study showed that FSG could increase the expression of Nrf2 and HO-1. FSG has been suggested to improve the intestinal barrier injury in CRF by mediating the Nrf2/HO-1 signaling pathway. MDA is an important marker of oxidative stress. Under stress conditions, the generation of oxygen-free radicals in the body increases, and oxygen-free radicals participate in the oxidative stress reaction and eventually generate the oxidative end-product MDA [51], as found in the present study. This study found that FSG could reduce the inflammatory state of T84 cells caused by urotoxin by reducing the COX-2 protein content. At the same time, FSG upregulated the expression of Nrf2 and HO-1, cleared the excessive accumulation of MDA in T84 cells, enhanced the cell antioxidant defense capability, and alleviated the effect of urotoxin on the oxidative stress of T84 cells. This study was the first to use network pharmacology to study the mechanism of FSG in treating CRF and conduct further *in vitro* experiments. The traditional Chinese medicine FSG reduced the inflammatory state of T84 cells induced by urotoxin through the Nrf2/HO-1 signaling pathway and decreased the damage to intestinal barrier function in CRF. This study had several shortcomings. Firstly, due to the limitations of the experimental conditions, only *in vitro* experiments were conducted, not *in vivo* experiments. Secondly, we did not detect the regulatory effect of urotoxin and FSG on Nrf2 protein expression in T84 cells. However, we did find that FSG upregulates the total Nrf2 protein expression. Therefore, the regulatory effect of FSG on HO-1 may be related to the promotion of Nrf2 into the nucleus. We will verify this hypothesis in a follow-up study.

## 6. Conclusion

FSG has a protective effect on urinary toxin-induced intestinal epithelial barrier injury in CRF, and its mechanism may be related to its upregulation of Nrf2/HO-1 signal transduction and thus the inhibition of tissue oxidative stress and inflammatory responses. Screening CRF targets and identifying the corresponding FSG components using a network pharmacological method is a practical strategy to explore the mechanism of FSG in improving gastrointestinal dysfunction in CRF.

## Figures and Tables

**Table 1:** Screening of core candidates.

Name	Average shortest path length	Closeness centrality	Clustering coefficient	Stress	Degree	Betweenness centrality	Topological coefficient
PTGS2	1.95402299	0	0.511765	16918	105	0.004564	0.464608
PTGS1	1.95402299	0	0.511765	16918	68	0.004564	0.464608
ADRB2	2.04597701	0	0.488764	9234	50	0.001962	0.51752
DPP4	1.96551724	0	0.508772	15470	45	0.003893	0.512909
RXRA	2.06896552	0	0.483333	6292	40	0.001063	0.649057
ADRA1B	2.04597701	0	0.488764	9234	38	0.001962	0.51752
PPARG	1.96551724	0	0.508772	15470	37	0.003893	0.512909
ESR1	1.96551724	0	0.508772	15470	35	0.003893	0.512909
F2	2.11494253	0	0.472826	4998	34	0.001416	0.461538

**Table 2:** GO analysis of candidate targets for FSG against CRF.

Category	Term	Count	%	*P* value	FDR
GOTERM_BP_DIRECT	GO: 0045944 ∼ positive regulation of transcription from RNA polymerase II promoter	15	0.116505	7.96*E − *07	0.001275
GOTERM_BP_DIRECT	GO: 0006954 ∼ inflammatory response	9	0.069903	3.04*E − *05	0.048613
GOTERM_BP_DIRECT	GO: 0010629 ∼ negative regulation of gene expression	8	0.062136	1.68*E − *07	2.70*E − *04
GOTERM_BP_DIRECT	GO: 0001666 ∼ response to hypoxia	7	0.054369	1.51*E − *06	0.002417
GOTERM_BP_DIRECT	GO: 0045766 ∼ positive regulation of angiogenesis	7	0.054369	4.47*E − *06	0.007153
GOTERM_BP_DIRECT	GO: 0043066 ∼ negative regulation of apoptotic process	7	0.054369	1.83*E − *03	2.882482
GOTERM_BP_DIRECT	GO: 0006351 ∼ transcription, DNA-templated	7	0.054369	1.21*E − *02	17.70194
GOTERM_BP_DIRECT	GO: 0006979 ∼ response to oxidative stress	6	0.046602	2.45*E − *05	0.03918
GOTERM_BP_DIRECT	GO: 0001525 ∼ angiogenesis	6	0.046602	3.80*E − *04	0.605925
GOTERM_BP_DIRECT	GO: 0010628 ∼ positive regulation of gene expression	6	0.046602	8.81*E − *04	1.40093
GOTERM_MF_DIRECT	GO: 0005125 ∼ cytokine activity	11	0.085437	6.08*E − *09	7.57*E − *06
GOTERM_MF_DIRECT	GO: 0020037 ∼ heme binding	8	0.062136	7.12*E − *06	0.008875
GOTERM_MF_DIRECT	GO: 0043565 ∼ sequence-specific DNA binding	8	0.062136	2.47*E − *03	3.030466
GOTERM_MF_DIRECT	GO: 0008083 ∼ growth factor activity	7	0.054369	1.00*E − *06	0.001248
GOTERM_MF_DIRECT	GO: 0004252 ∼ serine-type endopeptidase activity	6	0.046602	1.17*E − *03	1.447175
GOTERM_MF_DIRECT	GO: 0042802 ∼ identical protein binding	5	0.038835	1.21*E − *04	0.151175
GOTERM_MF_DIRECT	GO: 0002020 ∼ protease binding	4	0.031068	4.22*E − *04	0.524268
GOTERM_MF_DIRECT	GO: 0003707 ∼ steroid hormone receptor activity	4	0.031068	4.95*E − *03	6.000497
GOTERM_MF_DIRECT	GO: 0042803 ∼ protein homodimerization activity	4	0.031068	1.19*E − *02	13.83665
GOTERM_MF_DIRECT	GO: 0004937 ∼ alpha1-adrenergic receptor activity	3	0.023301	1.36*E − *04	0.169113
GOTERM_CC_DIRECT	Extracellular space	26	0.201942	3.25*E − *13	3.77*E − *10
GOTERM_CC_DIRECT	GO: 0070062 ∼ extracellular exosome	18	0.139806	7.97*E − *03	8.860488
GOTERM_CC_DIRECT	GO: 0005829 ∼ cytosol	16	0.124272	1.18*E − *05	0.013679
GOTERM_CC_DIRECT	GO: 0005794 ∼ Golgi apparatus	9	0.069903	1.16*E − *03	1.340888
GOTERM_CC_DIRECT	GO: 0005576 ∼ extracellular region	9	0.069903	2.31*E − *03	2.648495
GOTERM_CC_DIRECT	GO: 0045121 ∼ membrane raft	7	0.054369	6.90*E − *06	0.007999
GOTERM_CC_DIRECT	GO: 0009897 ∼ external side of plasma membrane	7	0.054369	6.50*E − *05	0.075398
GOTERM_CC_DIRECT	GO: 0009986 ∼ cell surface	7	0.054369	3.62*E − *03	4.113532
GOTERM_CC_DIRECT	GO: 0031012 ∼ extracellular matrix	6	0.046602	1.67*E − *04	0.19341
GOTERM_CC_DIRECT	GO: 0005925 ∼ focal adhesion	6	0.046602	1.08*E − *02	11.87756

**Table 3:** Enrichment analysis of candidate targets for FSG against CRF.

Category	Term	Count	%	*P* value	Genes
KEGG_PATHWAY	ssc05200: pathways in cancer	20	0.155339806	0.00	EGFR, IL6, PTGS2, RELA, MMP9, PPARG, MET, TP53, CXCL8, PTEN, MMP2, TGFB1, MMP1, PRKCB, CASP3, HIF1A, BCL2, VEGFA, NOS2, EGF
KEGG_PATHWAY	ssc05205: proteoglycans in cancer	15	0.116504854	0.00	EGFR, CAV1, TNF, MMP9, MET, TP53, ESR1, MMP2, TGFB1, PRKCB, KDR, CASP3, HIF1A, VEGFA, PLAU
KEGG_PATHWAY	ssc04151: PI3K-Akt signaling pathway	15	0.116504854	0.00	EGFR, IL4, IL6, RELA, MET, TP53, PTEN, KDR, BCL2, VEGFA, NOS3, EGF, INSR, IL2, SPP1
KEGG_PATHWAY	ssc04066: HIF-1 signaling pathway	14	0.108737864	0.00	EGFR, IL6, HIF1A, RELA, BCL2, IFNG, EDN1, VEGFA, NOS3, NOS2, EGF, INSR, PRKCB, TIMP1
KEGG_PATHWAY	ssc05144: malaria	12	0.093203883	0.00	VCAM1, ICAM1, IL6, CCL2, TNF, CD40LG, IFNG, MET, CXCL8, SELE, TGFB1, IL10
KEGG_PATHWAY	ssc04668: TNF signaling pathway	12	0.093203883	0.00	VCAM1, ICAM1, CASP3, IL6, CCL2, TNF, PTGS2, MMP9, RELA, EDN1, MMP3, SELE
KEGG_PATHWAY	ssc05206: micro-RNAs in cancer	12	0.093203883	0.00	EGFR, CASP3, PTGS2, BCL2, MMP9, VEGFA, MET, TP53, PTEN, SIRT1, PLAU, PRKCB
KEGG_PATHWAY	ssc05323: rheumatoid arthritis	11	0.085436893	0.00	ICAM1, IL6, CCL2, TNF, IFNG, VEGFA, CXCL8, MMP3, TGFB1, MMP1, IL1A
KEGG_PATHWAY	ssc05145: toxoplasmosis	11	0.085436893	0.00	CASP3, TNF, LDLR, CD40LG, BCL2, RELA, IFNG, NOS2, ALOX5, TGFB1, IL10
KEGG_PATHWAY	ssc05161: hepatitis B	11	0.085436893	0.00	CASP3, IL6, TNF, BCL2, MMP9, RELA, TP53, CXCL8, PTEN, TGFB1, PRKCB
KEGG_PATHWAY	ssc04060: cytokine-cytokine receptor interaction	11	0.085436893	0.00	IL4, IL6, CCL2, TNF, CD40LG, IFNG, CXCL8, TGFB1, IL1A, IL10, IL2
KEGG_PATHWAY	ssc05140: leishmaniasis	10	0.077669903	0.00	IL4, TNF, PTGS2, NCF1, RELA, IFNG, NOS2, TGFB1, IL1A, IL10
KEGG_PATHWAY	ssc05146: amoebiasis	10	0.077669903	0.00	CASP3, IL6, TNF, RELA, IFNG, CXCL8, NOS2, TGFB1, IL10, PRKCB
KEGG_PATHWAY	ssc05142: Chagas disease (American trypanosomiasis)	10	0.077669903	0.00	IL6, CCL2, TNF, RELA, IFNG, CXCL8, NOS2, TGFB1, IL10, IL2
KEGG_PATHWAY	ssc05202: transcriptional misregulation in cancer	10	0.077669903	0.00	IL6, MMP9, RELA, MET, PPARG, TP53, CXCL8, MPO, MMP3, PLAU
KEGG_PATHWAY	ssc05152: tuberculosis	10	0.077669903	0.00	CASP3, IL6, TNF, BCL2, RELA, IFNG, NOS2, TGFB1, IL1A, IL10
KEGG_PATHWAY	ssc04510: focal adhesion	10	0.077669903	0.00	EGFR, CAV1, BCL2, VEGFA, MET, EGF, PTEN, PRKCB, KDR, SPP1
KEGG_PATHWAY	ssc05321: inflammatory bowel disease (IBD)	9	0.069902913	0.00	IL4, IL6, TNF, RELA, IFNG, TGFB1, IL1A, IL10, IL2
KEGG_PATHWAY	ssc04064: NF-kappa B signaling pathway	9	0.069902913	0.00	VCAM1, ICAM1, TNF, PTGS2, CD40LG, BCL2, RELA, CXCL8, PLAU
KEGG_PATHWAY	ssc04068: FoxO signaling pathway	9	0.069902913	0.00	EGFR, IL6, CAT, EGF, INSR, PTEN, SIRT1, TGFB1, IL10

**Table 4:** Effects of various concentrations of uremic toxin and low-dose FSG-containing serum on T84 viability.

Group	*N*	OD value
Normal control group (C)	6	1.34 ± 0.60
42 mg dL^−1^ urea group (M1)	6	1.29 ± 0.90
72 mg dL^−1^ urea group (M2)	6	1.27 ± 0.63
144 mg dL^−1^ urea group (M3)	6	1.38 ± 0.85
42 mg dL^−1^ urea + 10% FSG low-dose serum (M1 + Y1)	6	1.28 ± 1.26
72 mg dL^−1^ urea + 10% FSG low-dose serum (M2 + Y1)	6	1.56 ± 0.14
144 mg dL^−1^ urea + 10% FSG low-dose serum (M3 + Y1)	6	1.39 ± 0.45

A490: mean ± SE. Compared to the normal control group, each group had no statistical significance (*P* > 0.05).

**Table 5:** Effects of various concentrations of uremic toxin and high-dose FSG-containing serum on the viability of T84 cells.

Group	*N*	OD value
Normal control group (C)	6	0.74 ± 0.06
42 mg dL^−1^ urea group (M1)	6	0.98 ± 0.08
72 mg dL^−1^ urea group (M2)	6	0.85 ± 1.35
144 mg dL^−1^ urea group (M3)	6	0.93 ± 1.05
42 mg dL^−1^ urea + 10% FSG high-dose serum (M1 + Y1)	6	1.54 ± 2.35
72 mg dL^−1^ urea + 10% FSG high-dose serum (M2 + Y1)	6	1.46 ± 1.61
144 mg dL^−1^ urea + 10% FSG high-dose serum (M3 + Y1)	6	1.40 ± 0.30

Compared to the normal control group, each group had no statistical significance (*P* > 0.05).

## Data Availability

All data generated or analyzed during this study are available from the corresponding author upon request.

## Abbreviations

CKD: Chronic kidney disease
FSG: Fushen Granule
CRF: Chronic renal failure
ZO-1: Zonula occludens-1
Nrf2: Nuclear factor E2-related factor 2
HO-1: Heme oxygenase 1
MDA: Malondialdehyde
COX-2: Cyclooxygenase-2
TCMSP: Chinese medicine system pharmacology database and analysis platform
GO: Gene ontology
KEGG: Kyoto encyclopedia of genes and genomes pathway.
